# Risk Factors and Cellular Differences in Heart Failure: The Key Role of Sex Hormones

**DOI:** 10.3390/biomedicines11113052

**Published:** 2023-11-14

**Authors:** Elvira Delcuratolo, Alberto Palazzuoli, Francesca Coppi, Anna Vittoria Mattioli, Paolo Severino, Francesco Tramonte, Francesco Fedele

**Affiliations:** 1 Specialization School of Cardiology, University of Siena, Viale Mario Bracci 16, 53100 Siena, Italy; elvira.delcuratolo1@gmail.com; 2 Cardiovascular Diseases Unit, Le Scotte Hospital, University of Siena, Viale Mario Bracci 16, 53100 Siena, Italy; molex829@gmail.com; 3 National Institute for Cardiovascular Research, Via Irnerio 48, 40126 Bologna, Italy; francesca.coppi@unimore.it (F.C.); annavittoria.mattioli@unimore.it (A.V.M.); francesco.fedele@uniroma1.it (F.F.); 4 Department of Clinical, Internal, Anesthesiology and Cardiovascular Sciences, Sapienza University of Rome, Viale del Policlinico, 155, 00161 Rome, Italy; paolo.severino@uniroma1.it

**Keywords:** heart failure phenotypes, sex differences, sex hormones, biomarkers, risk factors

## Abstract

Patients with heart failure are conventionally stratified into phenotypic groups based on their ejection fraction. The aim of this stratification is to improve disease management with a more targeted therapeutic approach. A further subdivision based on patient gender is justified. It is recognized that women are underrepresented in randomized controlled clinical trials, resulting in limited clinical and molecular differentiation between males and females. However, many observational studies show that the onset, development, and clinical course of the disease may substantially differ between the two sexes. According to the emerging concept of precision medicine, investigators should further explore the mechanisms responsible for the onset of heart failure due to sex differences. Indeed, the synergistic or opposing effects of sex hormones on the cardiovascular system and underlying heart failure mechanisms have not yet been clarified. Sex hormones, risk factors impact, and cardiovascular adaptations may be relevant for a better understanding of the intrinsic pathophysiological mechanisms in the two sexes. Despite the differences, treatment for HF is similar across the whole population, regardless of sex and gender. In our review, we describe the main differences in terms of cardiovascular dysfunction, risk factors, and cellular signaling modifications related to the hormonal pattern.

## 1. Introduction

Heart failure is one of the most common diseases of this century. Due to the increase in average survival and the growing incidence of cardiovascular risk factors and comorbidities, the number of people living with chronic heart failure (CHF) is increasing [1]. Therefore, it is mandatory to achieve a better understanding of this condition in order to define the optimal therapeutic management and diagnostic timeframe. It is essential to recognize elements related to sex differences, e.g., the incidence of particular risk factors and the related pathophysiological mechanisms [2]. The identification of specific patterns may lead to a more targeted and effective therapeutic approach. Indeed, the phenotypic manifestations of heart failure are different in men and women due to the different hormonal patterns, molecular signals, and cardiac structures in the two sexes. Although these mechanisms are well known in men, in women they are currently underestimated due to the underrepresentation of females in clinical trials and the lack of detailed sex-matching analysis [3,4]. The purpose of this review is to focus on a poorly understood topic, emphasizing the importance of recognizing and studying the mechanisms of heart failure in a segment of the population that has been inadequately represented and investigated in the past.

The applied selection criteria for manuscript construction were based on extensive literature research, selecting randomized studies published in English over the last 10 years in the PubMed/Medline database, including the following keywords: heart failure, gender, sex differences, risk factors, and hormones. Only extensive original papers and meta-analyses were included; abstracts, letters, and written comments were excluded.

## 2. Epidemiology

Based on the data analysis from the National Health and Nutritional Examination Surveys conducted between 2015 and 2018, the prevalence of heart failure in the population aged over 20 years is estimated to be around 6 million individuals. According to this registry, in the age range between 20 and 79 years, the prevalence of heart failure is higher in men than in women. However, in the older population, particularly among women, the prevalence increases [5,6]. Between 2014 and 2018, there was a comparable increase in the hospitalization rate for heart failure in both men and women [4]. Currently, the overall risk of developing heart failure is roughly similar for both sexes. In the Framingham Heart Study, it is estimated to be 21% in men and 20% in women at the age of 40, while in the Rotterdam Study, it is 33% in men and 29% in women at the age of 55 [7,8].

Despite a similar overall risk, women with heart failure, as identified by the Kansas City Cardiomyopathy Questionnaire, experience a greater burden of symptoms, an overall poorer health status, and lower quality of life [9]. Furthermore, significant epidemiological differences emerge when examining heart failure phenotypes. The Southwestern European community-based Epidemiology of Heart Failure and Learning (EPICA) study revealed that heart failure with preserved ejection fraction (HFpEF) is more prevalent in the female population, with a prevalence that increases with age (0% in men and 1% in women in the age group 25–49 years, increasing to 4–6% in men and 8–10% in women for individuals aged 80 or older) [10] (Table 1).

Even before the development of clinically overt heart failure, it has been observed that women are more predisposed to develop the disease at older ages, experiencing diastolic dysfunction, arterial stiffness, and coronary microvascular alterations. Microvascular dysfunction is probably the main determining factor in the pathogenesis of HFpEF in women [11,12,13]. The multinational multicenter study PROMIS-HFpEF (PRevalence Of MIcrovascular dysfunction in Heart Failure with Preserved Ejection Fraction), conducted on 202 patients with HFpEF, revealed that as many as 71% of women presented coronary microcirculation abnormalities [14].

However, according to various population studies, heart failure with reduced ejection fraction (HFrEF) is less common in women than in men, with different etiologies and comorbidities. In elderly women, the most frequent causes are hypertension and heart valve disease, while coronary artery disease is less frequent [15,16]. Women with HFrEF commonly exhibit symptoms such as dyspnea, jugular venous distention, and dependent edema [17]. Despite current discrepancies, the in-hospital mortality rate is similar between the two sexes, with a rate of 2.69% in women and 2.89% in men [18].

## 3. Risk Factors

Significant differences in the incidence and distribution of risk factors between men and women are well documented. This gap determines different characteristics in the frequency and types of heart failure (HF) in each sex. Furthermore, there are specific risk factors associated with each sex that deserve a detailed description. These differences in timing and prevalence contribute to the different phenotypic patterns that define the development and evolution of the disease, and the response to therapy (Table 2).

### 3.1. Diabetes Mellitus

Diabetes is a traditional risk factor for heart failure and has a stronger impact on women. Diabetes is associated with an approximately 5-fold increase in the risk of developing heart failure in women, compared to a 2.4-fold increased risk in men [19]. Furthermore, imaging techniques have revealed, in women, a higher rate of left ventricular remodeling characterized by increased left ventricular wall thickness and an elevated left ventricular mass index [20]. Diabetes appears to be implicated in the etiopathogenetic mechanisms of heart failure with preserved ejection fraction (HFpEF) through systemic vascular inflammation and endothelial dysfunction [21,22]. The production of free radicals and the reduced bioavailability of nitric oxide (NO), stemming from the biochemical and metabolic changes that occur in individuals with diabetes, lead to a decrease in the activity of guanylate monophosphate cyclase (GMPc) in cardiomyocytes. This results in reduced myocardial relaxation, an increased stimulus for hypertrophy, and increased wall stiffness [23,24]. The association between diabetes and HFpEF, as well as the relevance of diabetes as a risk factor in women, aligns with the greater prevalence of this phenotype in women.

### 3.2. Hypertension

Hypertension is a traditional risk factor associated with HF. Although the prevalence of arterial hypertension is similar in both sexes, according to the Framingham Heart Study, it appears to be more closely related to a greater incidence of heart failure in women (3-fold increased risk) than in men (2-fold increased risk) [25]. According to the results of the PARAMOUNT study (Prospective comparative of ARNI with ARB on Management Of heart failUre with preserved ejectioN section), women have greater arterial stiffness than men. This stiffness seems to be responsible for the development of cardiac hypertrophy and an increase in vascular resistance [26]. Additionally, increased blood pressure may lead to an increase in cardiac afterload, predisposing to the development of diastolic dysfunction. In addition to the structural changes in the heart muscle, hypertension can also lead to increased stiffness of the heart chambers, further contributing to diastolic dysfunction. Hypertension also determines a phenomenon of microvascular inflammation underlying the pathogenesis of HFpEF [21]. Interestingly, it has been suggested that there is a common pathophysiological pathway connecting preeclampsia, fetal growth retardation (FGR), and the subsequent development of heart failure with preserved ejection fraction (HFpEF) later in life [27]. This pathway involves various interconnected mechanisms. Preeclampsia increases chronic inflammation that may persist beyond pregnancy and contribute to the development of cardiovascular problems later in life. Preeclampsia is also associated with increased oxidative stress, potentially setting the stage for HFpEF. Some authors suggest that preeclampsia may accelerate vascular biological aging contributing to HFpEF [28].

These interconnected mechanisms collectively contribute to the development of HFpEF in individuals who have a history of preeclampsia and fetal growth retardation during pregnancy. This pathway is being investigated as a potential link; the exact mechanisms and the extent of their contribution to the development of HFpEF vary among individuals.

### 3.3. Obesity

Obesity is an important risk factor associated with the development of cardiovascular disease. Recently, the role of obesity in heart failure has been analyzed in depth, revealing that it is more frequently associated with HFpEF than with HFrEF. Furthermore, obesity has an important role as a greater risk factor in women than in men because it is associated with a sedentary lifestyle and reduced physical activity and also because of the inflammatory response associated with the visceral obesity of menopause [29,30,31]. The reason for the relationship between obesity and HFpEF appears to be due to some enhancement in its metabolic processes: insulin resistance is responsible for a systemic inflammatory state that leads to concentric cardiac remodeling [31,32]. Increased LDL deposition and oxidized fatty acid increase vascular oxidative stress, causing an endothelial damage drive leading to an increased vascular tone and microcirculatory vasoconstriction. Pre-menopausal women are relatively protected from these mechanisms thanks to the anti-inflammatory and antioxidant action of estrogens. However, with the decrease in circulating estrogen levels following menopause, there is a reduction of these protective mechanisms as well as a significant increase in visceral fat responsible for the increased risk of HFpEF [33,34,35]. Fat distribution is also crucial in determining cardiovascular risk; it is known that a visceral, central, or android-type distribution, more typical of men and post-menopausal women, is associated with a higher risk. Recent studies show that this type of adiposity is more harmful in women than in men, with a closer correlation and a higher cardiometabolic risk. Similarly, the gynoid distribution is more protective in men than in women, with a more significant decrease in risk in the former (90–84% lower in men versus 80–78% in women) [36].

Leptin and adiponectin are both hormones produced by adipose tissue, but they have different functions and effects on the body. Leptin is often referred to as the “satiety hormone” because its primary role is to regulate appetite and energy balance. Leptin also plays a role in regulating metabolism and energy expenditure. Adiponectin has various functions related to insulin sensitivity and anti-inflammatory effects. Adiponectin levels are generally higher in lean individuals and lower in those with obesity. Higher levels of adiponectin are associated with improved insulin sensitivity and metabolic health. Adiponectin levels tend to decrease with increasing body fat [30,33,35].

### 3.4. Tobacco Smoke

According to the First National Health and Nutrition Examination Survey (NHANES I) study, the risk of heart failure associated with cigarette smoking is higher for women than for men [37]. This higher risk to date has been offset by lower consumption by women, but this trend is currently changing, especially in industrialized countries [38,39].

A plausible reason is a change in the lifestyle of women who have taken on leadership positions, with the consequent stress due to the burden of responsibility. Moreover, women have a low perception of their cardiovascular risk and are more easily exposed to risk factors. Furthermore, with the widespread use of electronic smoking devices, the associated risk is likely to be underestimated, since little is known about the effects of new electronic and non-electronic smoking devices. This potential new risk factor must be further investigated [40].

### 3.5. Coronary Artery Disease

Coronary heart disease is the major risk factor for the development of HF, especially in industrialized countries [41]. It is now established that the incidence of macrovascular coronary artery disease is higher in men, leading more frequently to an HFrEF phenotype [42]. In recent studies, a close correlation has been seen between CAD and HFpEF, since it equally determines both systolic and diastolic dysfunction; the incidence of HFrEF and HFpEF related to CAD seems to be comparable [43]. This association appears to be stronger in males than in females for HFpEF; indeed, in the I-PRESERVE study, the cause of HFpEF was more frequently ischemic in men than in women (34% vs. 19%) [44].

A recent study examined disparities in all-cause mortality between women and men with HFrEF and the impact of the presence of CAD. Despite an overall lower mortality rate among female patients than among men, women without CAD had the lowest mortality, while women and men with CAD had a similar 10-year mortality risk. This study highlights that the presence of CAD has a significantly greater prognostic impact in women than in men with newly diagnosed HFrEF. These findings highlight the importance of implementing individualized risk assessment strategies for patients with newly diagnosed HFrEF, with particular attention to the increased mortality risk associated with the coexistence of CAD and HFrEF in women [45].

### 3.6. Anemia and Iron Deficiency

Anemia is associated with an increased risk of death and hospitalization in heart failure [46,47]. This is related to the activation of neurohormonal signals, implicated in oxidative metabolism and leading to systemic inflammation [44]. This hypothesis correlates well with the evidence from studies demonstrating a higher association between anemia and HFpEF [47,48,49]. It has been hypothesized that anemia and iron deficiency, known to be more common in women, could play a role in the increased predisposition for HFpEF [49,50].

### 3.7. Vitamin D Deficiency

Low levels of vitamin D (<20 ng/mL) seem to be predictive of an increase in cardiovascular mortality [51]. Although a direct correlation between vitamin D deficiency and the onset of heart failure has not yet been demonstrated, it has been ascertained that this molecule plays a key role in regulating many mechanisms associated with it. First, the VDRs regulate blood pressure by acting on the renin–angiotensin–aldosterone system; some studies on mice have shown how silencing of the VDR genes leads to an activation of the RAAS system with an increase in the onset of arterial hypertension and ventricular hypertrophy [52]. Furthermore, vitamin D deficiency seems to be implicated in the genesis of endothelial dysfunction through the reduction of nitric oxide production [53]. Adequate levels of vitamin D prevent the activation of the mechanisms that lead to the destabilization of atherosclerotic plaques: the downregulation of metalloproteinases MMP-2 and MMP-9 leads to a decrease in the levels of inflammatory cytokines and chemokines (IL-6, IL-12, interferon-γ, and TNF-α) and an increase in anti-inflammatory factors such as IL-10 [51,54,55]. Vitamin D appears to have an antithrombotic action through the regulation of thrombomodulin and tissue factor expression [56]. A large proportion of postmenopausal women (50–80%) is affected by vitamin D deficiency [57]. After the menopause transition, the cutaneous and hepatic production of vitamin D is reduced, as is its intestinal absorption [58,59]. In addition, estrogens regulate the hepatic production of vitamin D-binding protein (DBP) and albumin, to which vitamin D binds within the bloodstream [60]. In the post-menopausal period, the drop in estrogen levels is associated with a reduction in circulating vitamin D. The correlation between a lack of vitamin D and a drop in estrogen levels therefore makes post-menopausal women more exposed to cardiovascular alterations.

### 3.8. Anorexia

Anorexia nervosa (AN) is characterized by severe malnutrition and electrolyte imbalances and may be associated with different types of cardiovascular complications according to gender. In a nationwide registry, anorexia was associated with worse outcomes in males than in females: CV events, arrhythmia, and heart failure complications were more severe in males [61]. Most reports included small samples of women showing alterations linked to electrolyte imbalance, dysprotidemia, and iron deficiency. This condition facilitates arrhythmic complications, conduction abnormalities, autonomic dysfunction, and hypotension [62]. Additionally, interstitial fibrosis and loss of cross-striations and myofibrils occur in protein-calorie malnutrition, reducing myocardial mass and contractility. Finally, increased QT prolongation can occur as a consequence of electrolyte abnormalities [63]. QT prolongation is recognized as the main cause of sudden cardiac death in the AN population. Although anorexia is a condition much more frequent in females, cross-sectional studies comparing the two genders may contribute to a better understanding of the difference in the impact of this disease on males and females.

### 3.9. Sex-Specific Risk Factors in Women

Adverse outcomes of pregnancy that involve both the mother and the fetus have been identified as risk factors for cardiovascular disease [64]. Hypertension disorders during pregnancy, i.e., gestational hypertension, preeclampsia, eclampsia, as well as chronic hypertension, increase the risk of developing arterial hypertension, coronary artery disease, and heart failure up to 40 years later [64,65,66]. A persistent vascular dysfunction was found in women who had a hypertension-related disease during pregnancy [67].

Cardiovascular health before pregnancy has an impact on both adverse outcomes and future CV risk. A recent AHA statement highlighted that it is essential to assess cardiovascular health in young women and promote primary prevention by following the “8 essential” guidelines [27,68]. The “8 essential” framework includes eight key health factors: diet, physical activity, no smoking, body mass index, blood pressure, lipids, sleep health, and blood sugar. This framework provides a comprehensive approach to assessing and promoting cardiovascular health [69].

Gestational diabetes increases the risk of developing type 2 diabetes later in life and increases the risk of myocardial infarction and diabetic cardiomyopathy [31]. A large cohort study showed that women with gestational diabetes had a higher risk of developing heart failure (62% higher) [70,71].

Peripartum cardiomyopathy develops during the last months of pregnancy or in the months following delivery in women who have no other known cause of HF; it is a significant life-threatening cause of HF in women [72].

Breast cancer is associated with an increased risk of heart failure (HF). The radiotherapy and chemotherapy treatments commonly used after diagnosis are the primary causes [73].

Takotsubo syndrome is more frequent in women (female-male ratio of 9:1) due to the greater impact of emotional stress on cardiovascular events [42]. The hypothesized etiopathogenetic mechanism includes a dysfunction of the microcirculation of neurogenic origin [74].

## 4. Different Heart Failure Profiles between Sexes

The higher frequency of HFrEF in men and of HFpEF in women is a mirror of the different pathophysiological mechanisms underlying these conditions. Men are more predisposed to macrovascular alterations of the coronary arteries, a known etiological factor of HFrEF [2,4]. Women frequently present microcirculatory anomalies that seem to be at the basis of the development of HFpEF. As a result of a recent prospective multinational study, microvascular dysfunction was present in 75% of cases of HFpEF and was proportionally correlated to the increase in NT-proBNP [14]. On the basis of microvascular dysfunction, there is an alteration of the signal mediated by nitric oxide (NO). In women, the proinflammatory state induced by the comorbidities leads to a reduction in the activity of eNO synthase, a reduction in NO synthesis, and consequent dysregulation of arteriolar tone and an increase in blood pressure [75]. Furthermore, in post-menopause, the reduction in the level of estrogen leads to a further reduction in the production of NO, enhancing the aforementioned mechanism [11]. Women have a microvascular proinflammatory state due to a different immune pattern compared to men. Women present a higher level of proinflammatory cytokines and greater activity of CD4 and CD8 T cells, which support the onset of autoimmune diseases, often related to diastolic dysfunction [76,77].

This proinflammatory status is confirmed by higher levels of inflammatory biomarkers in women. In an analysis of healthy subjects from the Framingham Heart Study, women had a higher expression of markers such as CRP (C-reactive protein), hemopexin, and C2 [3]. Similarly, the Dallas Heart Study found higher levels of hsCRP (high-sensitivity CRP), D-dimer, and osteoprotegerin, and lower levels of IL (interleukin)-18 and phospholipase A2 [77] in women. These differences appear to be primarily X-linked: the human X chromosome includes multiple immune-related genes, such as IL-2 receptor-γ chain, IL-3 receptor-α chain, IL-9 receptor, IL-13 receptor-α chains, Toll-like receptor 7 (TLR7), TLR8, and IL-1 receptor-associated kinase [1,20,21]. Aside from a genetic basis, the proinflammatory state of women is influenced by the effects of estrogens on innate immunity and on adipose tissue deposition at the liver and abdominal levels [42,71]. Other differences between the two sexes are evident in ventricular and vascular mechanics. Women have anatomically smaller heart chambers than men, even after indexing by body surface area, with consequently lower stroke volumes; however, cardiac output is usually comparable in men and women thanks to a higher heart rate. Women are also affected by an increase in left ventricular wall stiffness, which increases with age [78]. With exercise, the lower chronotropic and contractile reserve of women, associated with the previously mentioned reduced stroke volumes, and higher ventricular rigidity, determine a reduced physical exercise tolerance, associated with a lower peak of oxygen consumption [79,80]. In women, vessels are smaller in size and have a greater wall stiffness, responsible for earlier wave reflection leading to an increase in afterload and blood pressure, diastolic dysfunction, and a concentric-type remodeling of the left ventricle [21,81,82]. The vessel wall thickness, not counterbalanced by an appropriate cardiac output, leads to an earlier loss of ventriculovascular coupling reserve during physical exercise in women, another key element of HFpEF [83,84]. Further, the pathophysiological feature of HFpEF is the association with pulmonary hypertension. Pulmonary hypertension is more frequent in women, due to a higher prevalence of mitral regurgitation and a greater susceptibility to idiopathic pulmonary hypertension [85,86].

## 5. Differences in Cellular and Endocrine Patterns

**Figure 1:**
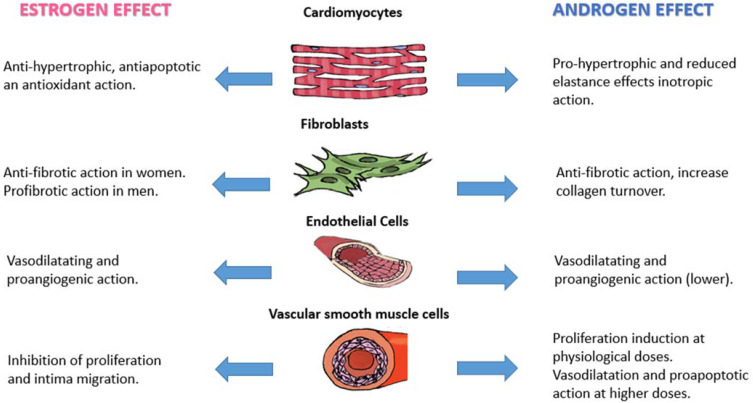
Effect of sex hormones on different cell types of the cardiovascular system.

The differences in the regulation of the cardiovascular system and the greater predisposition of the two sexes to the clinical manifestations described above can be justified by the action of sex hormones. Both estrogens and androgens play specific roles in some protective or predisposing pathways. The drop in the levels of these hormones that occurs with age determines alterations responsible for cardiovascular changes as well as for the development of chronic disease [87]. The action of sex hormones is exerted on various cellular targets through the interaction with different types of receptors, with the activation of intracellular patterns determining biochemical, electrolytic, genetic, or epigenetic modifications. A particular key role seems to be played through the expression of certain types of microRNAs regulated by sex hormones (Figure 1).

### 5.1. Cardiomyocytes

Estrogens, specifically 17β-estradiol (E2), exerting their action on cardiomyocytes, play a significant role in cardiovascular protection in women. Through the ERβ receptor and the activation of PI3K-stimulating MCIP1, calcineurin inhibition occurs. This protein is responsible for pro-hypertrophic and pro-fibrotic actions on cardiomyocytes; the anti-hypertrophic action mediated by the ERβ receptor is also carried out through transcriptional regulation. It determines a Ca+ flow inhibition leading to missed activation of Ca^2+^/calmodulin-dependent kinase II (CaMKII), which cannot phosphorylate HDAC4 and HDAC5. Subsequently, HDACs are free to silence MEF2 (myocyte-specific enhancer factor 2), which cannot promote hypertrophic gene expression [87,88]. By binding to the ERα receptor, on the other hand, they exert an antiapoptotic effect through the activation of the PI3K/Akt pathway [89]. Finally, they also have an antioxidant action: by inducing the expression of micro-RNA 22 through binding to ERα, they activate a cascade of events that leads to the reduction of radical oxygen species (ROS) production [90]. In post-menopause, the progressive decline in estrogen determines the loss of these protective mechanisms, with an increased risk of developing hypertrophy associated with increased ROS production and activity.

Androgens and testosterone, through their binding with the angiotensin receptors (AR), play an important action in the tropism of cardiomyocytes and their contractile function. An excess of testosterone is associated with an induction of hypertrophy and an increase in beta-adrenergic receptors and Ca^2+^ channels on the cytoplasmic membrane of cardiomyocytes, with a final effect of positive inotropism [91]. The decrease in testosterone levels with age determines a reduction in contractile function in men, which explains the higher incidence of HFrEF in this sex [92]. In elderly men, a greater rate of apoptosis of cardiomyocytes at the ventricular level has been reported, leading to an increase in the volume of the remaining cells [93,94].

### 5.2. Fibroblast

Fibrotic cardiac remodeling is more common in men than in women.

A greater quantity of metalloproteinase inhibitors and TGFβ-1 was detected in the walls of the left ventricle of men in response to a pressure overload [95]. This difference seems to depend on the action of E2 following binding to the ERβ receptor: by cAMP/PKA pathway, it determines an inhibition of TGF-β1 that cannot induce the transition of fibroblasts into myofibroblasts. Furthermore, this intracellular pathway prevents JNK activation, with consequent inhibition of SMAD2 and SMAD3 phosphorylation, which cannot express fibrotic genes, with a final anti-fibrotic action [96].

Even androgens seem to act on fibrosis inhibition [97]. Men develop this type of remodeling for the different actions of E2: the binding to the ERβ receptor activates a pro-fibrotic mechanism, with an increase in the expression of the genes coding for collagen I and III; the opposite response occurs in women [98].

### 5.3. Endothelial Cells and Vascular Smooth Muscle Cells

In this type of cell, estrogens enhance a vasodilation mechanism in women: by binding ERα, they activate the PI3K/Akt signal, which leads to the phosphorylation and activation of eNOS and, consequently, to the production of NO, with a vasodilating action [99]. Female hormones promote the expression of cyclooxygenase-1 and prostacyclin synthase enzymes, used for the production of prostacyclin (PGI2), another important vasodilating agent [100]. Furthermore, through the activation of the signal mediated by the vascular endothelial growth factor (VEGF), they stimulate the mechanism of angiogenesis [101]. Testosterone in men, through an indirect mechanism after its conversion into E2 by the aromatase enzyme, has a vasodilating action through the induction of NO production and favors the proliferation and migration of the precursors of endothelial cells [102,103]. Nevertheless, these mechanisms appear to be more efficient in women (excluding the post-menopausal age group) than in men, with evidence of a higher number of endothelial cell precursors and a more accentuated migratory function [104].

The action of estrogens on vascular smooth muscle cells is expressed through a reduction in proliferation and migration [105]. Female hormones are also able to induce the expression of the enzyme superoxide dismutase (SOD), reducing the levels of oxygen-free radicals [106]. The action of male hormones seems to be dose-dependent: at physiological doses, they stimulate their proliferation; at higher doses, they cause vasodilation and enhance progressive cell apoptosis [107].

## 6. Sex Hormones and Biomarkers in Cardiology Practice

The action of sex hormones exerted on the cells of the cardiovascular system seems to also affect the circulating levels of the main biomarkers used in cardiology clinical practice. It is therefore important to underline their action at this level for a more correct interpretation of the laboratory results according to the patient’s sex (Table 3).

### 6.1. Natriuretic Peptides

Natriuretic peptides are certainly the most used and recommended biomarkers for the diagnosis and follow-up of patients with heart failure, especially BNP and its precursor, NT-proBNP. Atrial NP (ANP) and type B NP (BNP), secreted by cardiomyocytes, appear to be the natriuretic peptides most involved in cardioprotection, antagonizing the hyperactivity of the renin-angiotensin system and promoting natriuresis and vasodilation [108,109]. Current guidelines recommend a universal NT-proBNP cut-off (125 ng/L non-acute) for the diagnosis of heart failure exclusion [110]. However, the different levels of this biomarker in the two sexes should be taken into account under normal conditions, with median levels between 45 and 70 ng/L in women and 25–40 ng/L in men [111]. Testosterone is responsible for the reduction of NP in males, probably through upregulation of the activity of their degrading enzyme, neprilysin [112].

However, there is conflicting evidence on the role of estrogens: it seems that they increase the release of NPs but at the same time the activity of neprilysin [113]. However, there is evidence that exogenous female hormone therapy is associated with a higher level of cardiac NP [114]. In patients with heart failure, this difference seems to disappear. This difference could depend on the different prevalence of the HF phenotype in the two sexes since generally lower mean levels of natriuretic peptides are found in HFpEF than in HFrEF [115,116]. Comparing the mean levels of natriuretic peptides in women and men stratified according to the ejection fraction, we can observe that they are always slightly higher in females: data confirmed by the Swedish HF registry conducted on 9847 patients, where the median NT-proBNP in patients with HFrEF was 2226 ng/L for men and 2543 ng/L for women, while in patients with HFpEF it was 1310 ng/L for men and 1598 ng/L for women [117].

### 6.2. Troponins

Similarly, troponins, biomarkers of myocardial damage, showed differences between men and women, with higher values in men [118]. In heart failure, there is an increase in troponin levels, which is more evident in men than in women: in a study of 504 patients with chronic heart failure, the median troponin levels in men were 23 ng/L versus a median of 18 ng/L in women [119]. This difference may depend on hormonal pathways: androgens seem to promote hypertrophy and apoptosis of cardiac cells and, through these mechanisms, may indirectly determine higher troponin levels [120].

### 6.3. Fibrosis Biomarkers: Galectin-3 and ST-2

Biomarkers indicative of fibrosis currently used for risk stratification in patients with heart failure are Galectin-3 and the soluble form of the receptor for interleukin 1 (ST2); in this context, once again, differences between the two sexes have been observed [121,122].

Galectin-3 (Gal-3) is a lectin secreted by activated macrophages with a pro-fibrotic action exerted on different organs [123]. In particular, it seems to be involved in the processes of cardiac remodeling and heart failure, in which an increase in its circulating levels can be appreciated, as the results of the FHS and FINRISK studies show. In healthy populations, baseline Gal-3 levels are higher in women (median value of 14.2 ng/L) than in men (median value of 12.8 ng/L) [124,125]. This difference, according to recent studies on the adult and pediatric populations, seems to be related to the quantity and distribution of fat mass; in this case, the different hormonal patterns of the two sexes would only play an indirect role [126].

Soluble suppressor of tumorigenicity 2 (sST2) is a soluble receptor belonging to the IL-1 receptor family. It seems to play a role in myocardial stress since, by binding to IL-33, it prevents its interaction with the corresponding transmembrane receptor, blocking its cardioprotective effects [127]. It is currently used as a prognostic biomarker in patients with heart failure, with a cut-off value of 35 μg/L [122]. However, a distinction should be made on the limit values based on the sex of the patient, since men have on average higher sST2 levels than women: in the healthy population, men have a mean value of 24.9 μg/L, women of 16.9 μg/L [128]. This difference is also confirmed in patients with heart failure [129]. The mechanisms underlying these findings are not yet clear, but it seems that in this case, sex hormones may also be involved; some studies have highlighted lower levels of ST2 in patients on exogenous estrogen therapy [130].

### 6.4. Osteopontin

Even in the levels of potential prognostic biomarkers currently under study, differences linked to the action of sexual hormones have been observed. This is the case of osteopontin. It is a protein of the cellular matrix that increases in heart failure [131]. It seems that osteopontin favors the processes of cardiac fibrosis and ventricular wall stiffness [132,133]. The levels of this glycoprotein are lower in women compared to men, and this difference is determined by the role of estrogen expression [133].

## 7. Limitations

Our paper reports the most recognized risk factors and endocrine differences related to sex, based on evidence in the literature. Further investigations are required to provide a more in-depth and exhaustive understanding of this topic. Additional risk factors may reveal new mechanisms that are not yet fully understood in the context of heart failure. Finally, the integration of traditional and new risk factors, proteomics, genomics, and metabolomics data may differentiate specific mechanisms and molecular signal dysfunction according to gender.

## 8. Conclusions

Women are underrepresented in HF clinical trials, and most of the findings resulting from interventional studies derive from the male population, which represents almost 70% of all HF patients. Nevertheless, women are an increasing population, particularly among the elderly and in HFpEF subsets. Current sex-specific aspects deserve a more individualized approach and therapeutic target. Most sex differences can be attributed to the different endocrine profiles responsible for multiple functional and morphological HF scenarios. Specific investigations focused on different HF profiles and therapeutic responses are a challenge for future research.

Today, the guidelines on heart failure only include brief comments on sex differences and the impact of hormones in the diagnostic and therapeutic evaluation of patients with heart failure [134]. This is partly due to the effectiveness of new drugs that reduce re-hospitalizations and complications of heart failure and have focused attention on treatment. However, the increasing evidence on the influence of hormones on the development of heart failure will certainly lead to a focus on sex differences in future guidelines.

Future research may focus on sex-specific differentiation by conducting a meticulous laboratory and molecular mechanistic investigation to clarify specific pathophysiological pathways according to gender.

Finally, a detailed assessment of risk factors and intrinsic cellular mechanisms is required to determine the optimal diagnostic evaluation and the best-tailored treatment. 

## Figures and Tables

**Table 1:** Summary of epidemiological data on heart failure in men and women from major studies in the scientific literature.

Epidemiological Data	Men	Women
Framingham Heart Study	The risk of developing heart failure is 21% at age 40 years	The risk of developing heart failure is 20% at age 40 years
Rotterdam Study	The risk of developing heart failure is 33% at age 55 years	The risk of developing heart failure is 29% at age 55 years
Southwestern European community-based Epidemiology of Heart Failure and Learning (EPICA) study	The prevalence of heart failure with preserved ejection fraction is 0% in the 25–49 age group, and increases to 4–6% in men aged 80 or older	The prevalence of heart failure with preserved ejection fraction is 1% in the 25–49 age group, and increases to 8–10% in women aged 80 or older

**Table 2:** Differences in main risk factors for heart failure between sexes.

Risk Factor	Men	Women
Diabetes mellitus	2.4-fold increased risk of HF	5-fold increased risk of HF
Hypertension	2-fold increased risk of HF	3-fold increased risk oh HF
Obesity	Lower association with male sex and HFrEF	Greater association with female sex and HFpEF
Tobacco smoke	Higher consumption but lower correlation (45% increased risk)	Lower consumption (increasing) but higher correlation (88% increased risk)
Coronary artery disease	Macrovascular disease more associated with both HFrEF and HFpEF	Less frequent, more often microvascular dysfunction
Anemia and iron deficiency	Less frequent	More frequent and associated with HFpEF
Vitamin D deficiency	Less frequent	More frequent and associated with higher cardiovascular mortality
Anorexia nervosa	Higher association with cardiovascular events	More frequent

**Table 3:** Effect of sex hormones on heart failure biomarkers. (1) NT-proBNP: N-terminal pro-B-type Natriuretic Peptide, the most common biomarker used for heart failure diagnosis and follow-up. (2) Troponin: the main marker of cardiomyocyte damage. (3) Galectin-3: fibrotic process biomarker. (4) s-ST-2: soluble Suppressor of Tumorigenicity 2, a soluble IL-1 receptor-like used as a prognostic biomarker in heart failure. (5) Osteopontin: a new potential prognostic biomarker in heart failure, indicative of fibrosis.

Biomarkers	Androgens	Estrogens
NT-proBNP (1)	Lower basal level in men due to probable upregulation of neprilysin by testosterone	• Higher basal level in women • Exogenous estrogen therapy associated with a higher level
Troponin (2)	Higher basal values and greater increase in male with HF due to the prohypertrophic and proapoptotic hormonal action	Lower baseline values and smaller increase in women with HF due to the antiapoptotic action of estrogens
Galectin-3 (3)	Reduced peripheral fat accumulation with lower level in men	Greater fat mass due to estrogen with higher level in women
sST-2 (4)	Higher mean values in men both in health and in HF	• Lower mean values in women both in health and in HF • Lower level during exogenous estrogen therapy
Osteopontin (5)	Higher level in men	Estrogen inhibition with lower level in women

## Abbreviations

AHA	American Heart Association
Akt	Ak strain transforming
AN	Anorexia Nervosa
AR	Androgen Receptor
BNP	B-type Natriuretic Peptide
CAD	Coronary Artery Disease
CaMKII	Ca^2+^/calmodulin-dependent kinase II
cAMP	Cyclic Adenosine Monophosphate
CRP	C-Reactive Protein
CV	Cardiovascular
DBP	Vitamin D Binding Protein
E2	17β-Estradiol
eNOS	Endothelial Nitric Oxide Synthase
EPICA	Southwestern European community-based Epidemiology of Heart Failure and Learning
ER	Estrogen Receptor
FHS	Framingham Heart Study
Gal-3	Galectin 3
GMPc	Guanylate MonoPhosphate cyclase
HDACs	Histone deacetylases
HF	Heart Failure
HFpEF	Heart Failure with preserved Ejection Fraction
HFrEF	Heart Failure with reduced Ejection Fraction
I-PRESERVE	Irbesartan in Heart Failure with Preserved Ejection Fraction
IL	Interleukin
LDL	Low-Density Lipoprotein
JNK	c-Jun N-terminal kinase
MCIP1	Myocyte-enriched Calcineurin-Interacting Protein
MEF2	Myocyte-specific Enhancer Factor 2
MMP	Matrix Metalloproteinase
NHANES I	First National Health and Nutrition Examination Survey
NO	Nitric Oxide
NP	Natriuretic Peptide
NT-proBNP	N-terminal pro-B-type Natriuretic Peptide
PARAMOUNT	Prospective comparative of ARNI with ARB on Management Of heart failure Ure with preserved ejectioN section
PI3K	Phosphoinositide 3-kinases
PGI2	Prostaglandin I2
PKA	Protein Kinase A
PROMIS-HFpEF	PRevalence Of MIcrovascular dysfunction in Heart Failure with Preserved Ejection Fraction
RAAS	Renin-Angiotensin-Aldosterone System
ROS	Radical Oxygen Species
SMAD	Suppressor of Mothers against Decapentaplegic
SOD	Superoxide Dismutase
sST2	Soluble Suppressor of Tumorigenicity 2
TGF	Transforming Growth Factor
TLR	Toll-like receptor
TNF	Tumor Necrosis Factor
VDR	Vitamin D Receptor
VEGF	Vascular Endothelial Growth Factor
